# Molecular Cloning, Expression and Macrophage Activation of an Immunoregulatory Protein from *Cordyceps militaris*

**DOI:** 10.3390/molecules26237107

**Published:** 2021-11-24

**Authors:** Qing Yang, Binmei Jia, Xiaomei Liu, Jialing Fang, Luyang Zhao, Lin Xu, Min Fang, Zhiyong Gong, Hui Sun

**Affiliations:** 1 Key Laboratory for Deep Processing of Major Grain and Oil of Ministry of Education, Wuhan Polytechnic University, Wuhan 430023, China; 15926352256@163.com (Q.Y.); mygroup001@163.com (B.J.); xulinlin2008@126.com (L.X.); fangmin0227@126.com (M.F.); gongwhqg@163.com (Z.G.); 2 College of Life Sciences, Wuhan University, Wuhan 430071, China; 2018202040152@whu.edu.cn (X.L.); 2015301060057@whu.edu.cn (J.F.); zhaoly19@mails.tsinghua.edu.cn (L.Z.); 3 Hubei Province Key Laboratory of Allergy and Immunology, Wuhan University, Wuhan 430071, China

**Keywords:** *Cordyceps militaris*, recombinant CMIP, macrophage

## Abstract

Protein components of *C. militaris* have been reported to possess various biological activities. In our previous research, a *Cordyceps militaris*-derived immunoregulatory protein (CMIP) was naturally isolated and showed the activity of inhibiting the metastasis of breast cancer cells. This study aimed to obtain recombinant CMIP (rCMIP) using recombinant expression and elucidate its ability to activate macrophages. Recombinant CMIP showed one band at approximately 15 kDa or 30 kDa, or two bands at 15 kDa and 30 kDa, under different denaturation conditions of electrophoresis. The cell binding assay showed that rCMIP selectively binds to the surface of macrophages. After adhesion, it did not induce the apoptosis of RAW 264.7 cells, but promoted their proliferation. Moreover, rCMIP significantly induced the expression of M1 macrophage polarization-related molecules. The mean fluorescence intensity (MFI) of CD 86 was enhanced by 2.1-fold and 3.2-fold under 0.64 μM and 1.6 μM of rCMIP treatment, respectively. Cytokines typically expressed in M1 macrophages, such as TNF-α, iNOS, IL-6, CCL 4, CCL 5 and CXCL 10, were also considerably induced by rCMIP, while the expression of cytokines in typical M2 macrophages, like Arg-1, CCL17 and CCL22, were not changed or slightly decreased. Under rCMIP treatment, the release of NO was also appreciably induced. In the present study, we reported cloning, expression and functional characterization of rCMIP, which was naturally isolated from the fruiting body of *C. militaris* in our previous study. The data imply that rCMIP possesses immunomodulatory activity in macrophages.

## 1. Introduction

Mushrooms are considered nutritional foods and a precious source of health-promoting agents, especially in Asian countries [1]. Proteins, one of the most important active ingredients in mushrooms, have been extensively studied for their pharmacological activities, including anti-tumor, antioxidant and immuno-regulatory activities [2,3,4]. Among these proteins, fungal immunomodulatory proteins (FIP) attract extensive attention worldwide for their potential application as an immunomodulatory supplement [5,6].

*Cordyceps militaris* (*C. militaris*) is one of the most famous functional mushrooms as it contains multiple active ingredients, like polysaccharides, mannitol and proteins [7,8,9,10]. Various pharmacological activities, such as anti-cancer, anti-inflammatory and immunoregulatory activities, of *Cordyceps militaris* have been reported extensively [11]. Many of these pharmacological activities are attributed to the protein components from *C. militaris* [12]. The anti-tumor activities of proteins from *C. militaris,* whether through their direct toxicity to tumor cells or immunoregulatory activity, have been especially widely reported [13,14]. All the studies indicated that *C. militaris*-derived proteins showed good application prospects as a health supplement.

Macrophages play a vital role in the immune response [15]. After activation, they exert immunomodulatory effects by releasing a wide array of immune molecules, like cytokines and chemokines [16]. According to the activation signal and differentiated phenotypes, macrophages have been divided into two groups: classically active macrophages (M1) and alternatively activated macrophages (M2) [17]. M1 macrophages produce a series of cytokines and immune effector molecules, such as NO, IL-6 and chemokines, to exert immune response [18]. M2 macrophages are able to secrete high amounts of anti-inflammatory cytokines, like TGF-βand Arg-1 [18]. The balance of macrophage M1–M2 polarization plays a vital role in tumor development [19,20]. Therefore, regulating the polarization of macrophages is a promising direction of tumor immunotherapy [21].

In our previous study, we reported a novel immunoregulatory protein from the fruiting body of *C. militaris*, which possesses anti-metastasis activity against breast tumors [14]. In the present study, recombinant CMIP obtained through prokaryotic expression was used to investigate its ability to induce macrophage activation. Cell binding selectivity and pro-inflammatory and anti-inflammatory mediators were detected to elucidate the effect of rCMIP on the polarization of macrophages. This study provides vital data, which support the potential application of rCMIP as a macrophage-specific activator.

## 2. Results

### 2.1. Cloning, Expression and Purification of Recombinant CMIP

**Figure 1:**
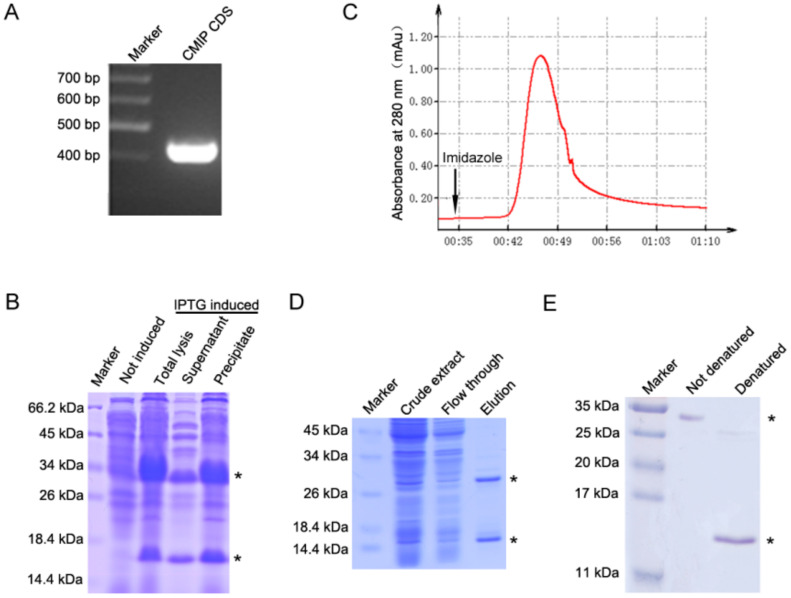
Cloning, expression and purification of rCMIP. (**A**) Total RNA was isolated from the fruiting body of *C. militaris* and amplified by reverse transcript PCR. The CMIP encoding sequence (414 bp) was amplified with designed primer and detected by agarose gel. (**B**) The rCMIP coding sequence was cloned into pET-30a plasmid, and then transformed into *E. coli* BL21 cells. Two bands appeared at ~30 kDa and ~15 kDa (marked with asterisks) after 0.5 mM IPTG induction for 16 h. (**C**) rCMIP was eluted as a single peak with elution buffer under 280 nm UV detection. (**D**) The elution of rCMIP produced two bands, corresponding to ~30 kDa and ~15 kDa (marked with asterisks) in molecular mass on SDS–PAGE. (**E**) rCMIP was detected by SDS–PAGE under completely denatured and non-denatured conditions of electrophoresis. A single bond appeared at ~30 kDa and another at ~15 kDa (marked with asterisks) by non-denaturing and denaturing treatment, respectively. The asterisks in (**B**,**D**,**E**) indicated the bands of targeted protein.

In our previous study, a novel immunoregulatory protein, namely CMIP, was isolated from the fruiting body of *C. militaris.* It was reported to possess anti-metastasis activity against 4T1 carcinoma. The gene of CMIP has been identified to be CCM_01955 by LC–MS/MS [14]. The nucleotide sequence of CCM_01955 and the amino acid sequence it encoded are provided in Supplementary Figure S1. In this study, the gene of CMIP (CCM_01955) was cloned using specifically designed primers. Agarose gel electrophoresis showed that the PCR amplification with cDNA and specific primers yielded the target gene with 414 bp (designated as rCMIP) (Figure 1A).

The expression of recombinant CMIP (rCMIP) was achieved by inducing it with different concentrations of IPTG, and by using various incubation times and temperatures (data not shown). Ultimately, the optimum expression condition was 0.5 mM of IPTG at 37 °C with 5 h of induction. The expression of rCMIP detected by SDS–PAGE protein electrophoresis revealed that two thick bands, one band at about 15 kDa and the other band at 30 kDa, were induced, compared to the non-IPTG-induced sample (Figure 1B). The induced bond at 15 kDa is a monomer of rCMIP and the induced bond at 30 kDa suggested that the recombinant CMIP may have existed as a dimer.

As the C-terminal of recombinant CMIP contains a His tag, a Ni-NTA affinity chromatographic column was used to purify the target protein. Under 280 nm UV detection, the recombinant CMIP (rCMIP) was eluted as a single peak with an elution buffer (Figure 1C). The elution of rCMIP produced two bands, corresponding to about 30 kDa and 15 kDa in molecular mass on SDS–PAGE (Figure 1D). To check that the rCMIP is in dimer form, the elution samples with complete denaturing treatment and non-denaturing treatment were detected by SDS–PAGE. The results showed that just one clear band, about 30 kDa, appeared under non-denaturing conditions and one clear band, about 15 kDa, appeared under complete denaturing conditions (Figure 1E).

### 2.2. Cell Binding Specificity of Recombinant CMIP

**Figure 2:**
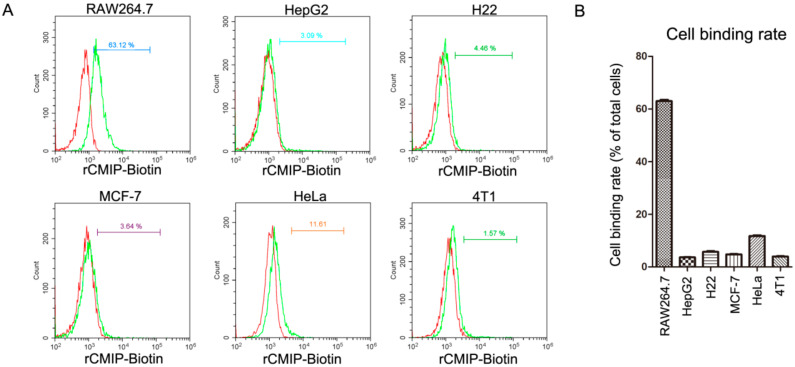
Cell binding selectivity of rCMIP. (**A**) RAW 264.7, HepG2, H22, MCF-7, HeLa and 4T1 cells (1 × 10^6^) were incubated with biotinylated rCMIP (2.5 μM) at 4 °C for 2 h. After washing, cells were incubated with streptavidin-FITC (SA-FITC) and analyzed by flow cytometry. Results are representative of three independent assays. (**B**) The cell labeling ratio quantitation of the tested cells from the flow cytometry results. All the data are presented with means ± SD (*n* = 3).

In the cell binding assay, among the six detected cells, rCMIP showed a specific binding to RAW 264.7 cells, but not to the other tested cells (Figure 2A). After being incubated with 2.5 μM of rCMIP, about 63% of the RAW 264.7 cells were labeled by rCMIP. However, the labeling ratios of the other five tested cells (HeLa, MCF-7, HepG2, H22 and 4T1) were all <12% (Figure 2B). The naturally purified CMIP also showed specific binding to RAW 264.7 cells rather than the other tested cells, which was consistent with the recombinant CMIP (Supplementary Figure S2). The consistency of the cell binding assay results from rCMIP and the naturally purified CMIP confirmed that the activity of rCMIP was the same as the naturally purified CMIP. The cell binding specificity of rCMIP to RAW 264.7 suggested that it has the potential to act as an activator of macrophages.

### 2.3. Effect of Recombinant CMIP on Cell Growth of Macrophages

**Figure 3:**
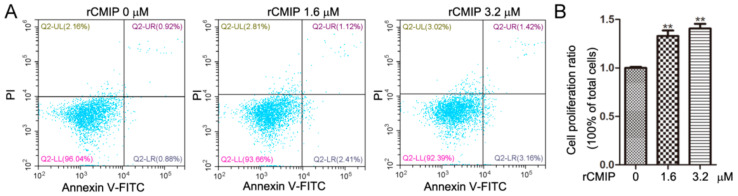
Effect of rCMIP on the growth of RAW 264.7 cells. (**A**) RAW 264.7 cells were treated with 1.6 μM and 3.2 μM of rCMIP for 24 h. Cells treated with PBS acted as the control. The apoptotic RAW 264.7 cells gated on Annexin V+/PI- were determined by Annexin V/PI double staining and a flow cytometric assay. (**B**) Cell proliferation of RAW 264.7 cells treated by rCMIP (0 μM, 1.6 μM and 3.2 μM) for 24 h was analyzed by CCK-8 assay. The data shown are the means ± SD (** p < 0.01) of three independent experiments.

The results of cell binding assay showed that rCMIP treatment even at 3.2 μM did not induce apoptosis of RAW 264.7 compared to the control (Figure 3A). Instead, rCMIP treatment (1.6 μM and 3.2 μM) significantly promoted the proliferation of RAW 264.7 macrophage cells compared to the untreated cells (Figure 3B). These results indicated that not only did rCMIP not cause apoptosis, but it also promoted the growth of RAW 264.7 cells.

### 2.4. rCMIP-Induced Macrophages Polarized to M1 Phenotype

**Figure 4:**
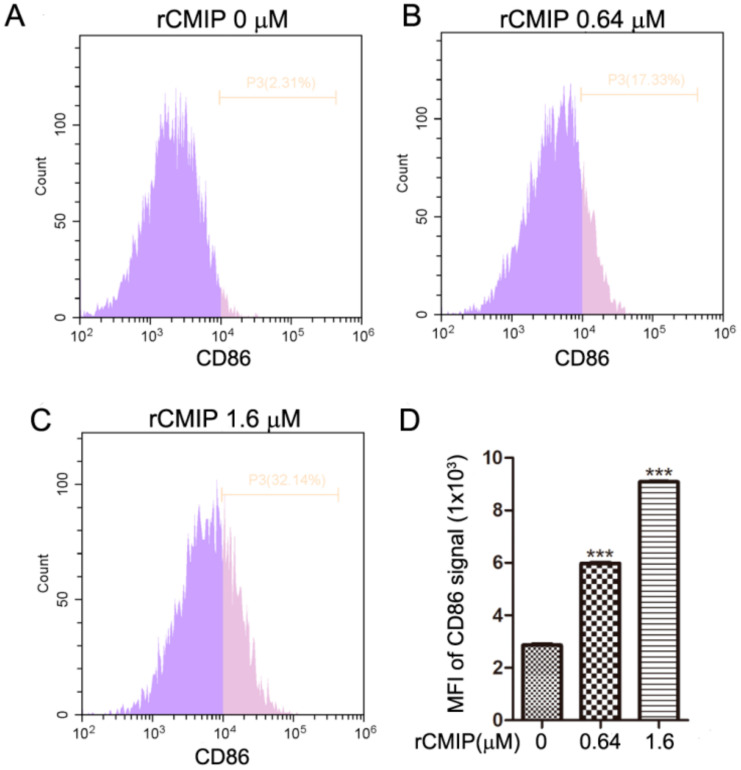
(**A**–**C**) rCMIP regulates the expression of CD86 in RAW 264.7 cells. RAW 264.7 cells were treated with 0.64 μM and 1.6 μM of rCMIP for 24 h. Cells treated with PBS acted as the control. The CD86 expression was detected by flow cytometry assay. (**D**) Histogram statistics of the data from the cytometry assay. Results are means ± SD (*n* = 3). *** *p* < 0.001.

To further investigate the effect of rCMIP on macrophages, expressions of CD86 molecule, cytokines and NO production were detected after rCMIP treatment. As shown in Figure 4A–C, compared to the untreated group, rCMIP (0.64 μM and 1.6 μM) considerably induced the expression of CD86 on the surface of RAW 264.7 cells. The MFI (mean fluorescence intensity) of CD86 signals treated with 0.64 μM and 1.6 μM of rCMIP were 5.9 × 10^3^ and 9.0 × 10^3^, respectively, which were significantly higher than the control group (2.8 × 10^3^) (Figure 4D).

**Figure 5:**
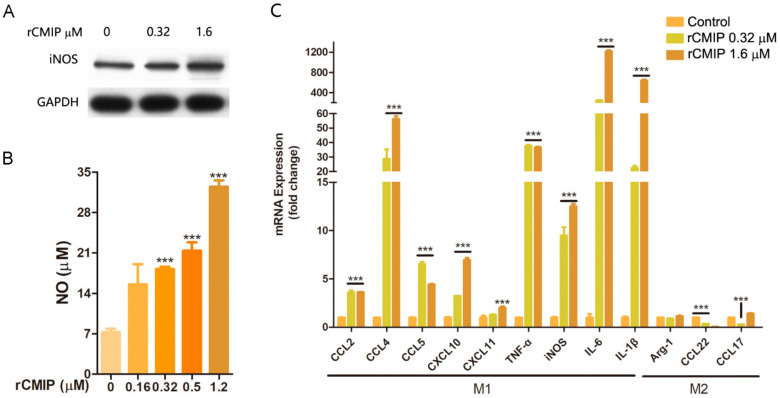
Effects of rCMIP on the polarization of RAW 264.7 cells. (**A**) iNOS protein expression of RAW 264.7 cells treated by rCMIP (0 μM, 0.32μM and 1.6μM) was analyzed by Western blot. (**B**) The release of NO in response to rCMIP (0 μM, 0.16 μM, 0.32 μM, 0.5 μM and 1.2 μM) treatment. (**C**) RAW 264.7 cells were treated with rCMIP (0 μM, 0.32 μM and 1.6 μM) for 3 h. The mRNA expression fold changes of M1 polarization mRNA (CCL2, CCL4, CCL5, CXCL11, CXCL10, IL-1β, TNF-α, iNOS and IL-6) and M2 polarization mRNA (Arg-1, CCL22 and CCL17) were tested by q-PCR assay. Data were expressed as the means ± SD (*n* = 3) (*** p < 0.001).

The results of the NO-releasing assay showed that rCMIP dramatically increased the release of NO. The amounts of NO released by RAW 264.7 cells were about 21.41 μM and 32.48 μM for treatment with 0.5 μM and 1.2 μM of rCMIP, respectively. These were remarkably higher than the control (~7.21 μM) (Figure 5B). The results of the Western blot showed that rCMIP treatment (0.32 μM and 1.6 μM) considerably increased the expression of iNOS of RAW 264.7 cells (Figure 5A). The increased expression of iNOS by rCMIP treatment was considered to be closely related to the increased release of NO.

To find out how rCMIP affected the expression of cytokines in macrophages, we treated RAW 264.7 cells with rCMIP (0.32 μM and 1.6 μM) for 3 h and examined their cytokine mRNA expression levels by q-PCR. From the results, we found that the mRNA expressions of the typical cytokines of M1 macrophages, including chemokines (CCL 2, CCL 4, CCL 5, CXCL 10 and CXCL 11), TNF-α, IL-6, IL-1β and iNOS were all remarkably up-regulated in rCMIP-treated RAW 264.7 cells. The mRNA expression of IL-6 increased by 243-fold and 1216-fold when treated with 0.32 μM and 1.6 μM of rCMIP, respectively. However, the cytokines typically expressed in M2 macrophages, like Arg-1, CCL22 and CCL17, were not changed or even slightly decreased after rCMIP treatment (Figure 5C). These results indicated that rCMIP has the potential to induce the M1 polarization of macrophages.

## 3. Discussion

*C. militaris* is renowned as a functional food for its immuno-regulatory, anti-tumor and nutritional benefits [22]. Protein is a very important ingredient of *C. militaris*. Some *C. militaris*-derived proteins, like CML, *C. militaris* HA and cordymin, have been isolated through a series of chromatographic separation methods [23,24,25]. The complex purification procedure seriously impedes the application of these active proteins. So, a recombinant expression of a naturally active protein, of which the quality can be consistent, is a suitable substitute for a naturally purified one [26,27]. In our previous study, we purified a novel *C. militaris*-derived immunoregulatory protein (CMIP) through a three-step chromatographic procedure and elucidated its anti-metastasis activity [14]. To further explore the effects of CMIP on the polarization of macrophages, which may be the underlying mechanism responsible for the anti-metastasis activity of *C. militaris*, we chose to clone the gene of CMIP and express it in prokaryotic cells (Figure 1A,B). The rCMIP was eluted as a single peak with an elution buffer under the 280 mm UV detection (Figure 1C). To some extent, the purity of rCMIP was guaranteed by expression and purification in vitro. Compared with the method of natural separation and purification of CMIP, the purification process of recombinant CMIP has been greatly simplified.

Fungal proteins with immunoregulatory activity get significant attention because they are potential biological ingredients or functional food ingredients, and can be produced in vitro by biotechnological methods [5]. Extensive studies have hitherto shown that fungal-derived immunoregulatory proteins possess anti-tumor activities, including the inhibition of tumor cell proliferation, the induction of apoptosis and autophagy and the inhibition of cell invasion and migration [5]. FIP-glu1(LZ-8), a typical FIP derived from *Ganoderma lucidum*, showed extensive tumor suppression effects on many tumor cells, like HCC, gastric cancer and lung cancer cells [28,29,30]. In our previous study, naturally purified CMIP was shown to prevent breast cancer cells from migrating to the lungs in vivo. In vitro, it did not affect the growth of 4T1 breast cancer cells, however the CMIP macrophage-conditional medium inhibited the cells’ growth [14]. In the present study, a cell binding assay showed that rCMIP did not bind to 4T1 cells and other tested tumor cells (Figure 2). Since there was no combination, there was certainly no signal transmission. This may explain why CMIP did not affect the growth of 4T1 cells as reported by our previous research. Activation of the host immune response is another critical anti-tumor mechanism of fungal-derived proteins [5]. Cytokines are crucial in the induction and regulation of the immune response at the cellular level. It was found that a *Pleurotus citrinopileatus*-derived protein, PCP-3A, has been shown to inhibit the growth of U937 cells by inducing the secretion of cytokines (IL-2, TNF-α and IFN-γ) by human mononuclear cells [31]. A novel *Maitake* protein was reported to exert anti-tumor activity on tumor-bearing mice by activating natural killer cells and dendritic cells [32]. In fact, many fungal-derived proteins exert their anti-tumor activity in this way. In our previous study, naturally prepared CMIP could active macrophages to release inflammatory cytokines. It is macrophage CMIP-conditioned media, not CMIP, that showed anti-proliferation effects on 4T1 cells. These results suggested that CMIP-induced macrophage activation has participated in the anti-tumor metastasis of CMIP [14]. In the cell binding assay of the present study, both rCMIP and naturally purified CMIP showed selective binding to macrophage RAW 264.7 cells but not to the other tested tumor cells (Figure 2 and Supplementary Figure S2). The binding of CMIP to macrophages met the prerequisite for the activation of macrophages. After binding, CMIP did not induce the apoptosis of macrophage RAW 264.7 cells (Figure 3A), but remarkably promoted their proliferation in a dose-dependent manner (Figure 3B). These results provided important clues for rCMIP as a macrophage activator.

Macrophages play an important role in host defense. Producing cytotoxic and inflammatory mediators (like NO, TNF-α, IL-6 and IL-1β) is an important way for macrophages to exert their nonspecific immunity. The activation of macrophages can be induced by a series of mushroom-derived substances, such as polysaccharides and proteins. After activation, macrophages may produce a series of pro-inflammatory molecules, like NO, TNF-α, CCL2, IL-1β, IL-6, CXCL 10 and CD 86. Macrophages with these properties are classically activated macrophages (proinflammation, M1) [33,34,35]. Our results implied that rCMIP played a predominant role in the expression of pro-inflammatory genes of macrophages, including CCL2, CCL4, CCL5, CXCL10, CXCL11, TNF-α, iNOS, IL-1β and IL-6 (Figure 5C). TNF-α is a pro-inflammatory cytokine and plays an essential role in the immune response. It has been used in the treatment of locally advanced soft tissue sarcomas, metastatic melanomas and other unresectable tumors [36]. In this study, the TNF-α mRNA expression of rCMIP-treated macrophages was > 35 times higher than the untreated cells (Figure 5C). The CC chemokine and CXC chemokine families are involved in the attraction and activation of monocytes and lymphocytes [37]. After rCMIP treatment, the mRNA expressions of CC chemokines (CCL2, CCL4 and CCL5) and CXC chemokines (CXCL 10 and CXCL 11) were also obviously increased. Among them, the mRNA expression of CCL 4 that had been enhanced by about 56-fold when treated with 1.6 μM of rCMIP was the most prominent (Figure 5C). iNOS-dependent NO released from activated macrophages is involved in many diseases, including cancer, as a cytotoxic mediator [38]. In rCMIP-treated macrophage RAW 264.7 cells, the protein expression of iNOS and the release of NO were both significantly up-regulated compared to the control group (Figure 5A,B). The correlation implicated that there existed an iNOS-dependent NO release in rCMIP-treated macrophages. The protein expression of CD 86, a typical molecule expressed on the surface of M1 macrophages, was also promoted by rCMIP in a dose-dependent manner (Figure 4). Alternatively activated macrophages (anti-inflammation macrophages, M2) were shown to inhibit inflammation by producing inflammatory molecules, such as CCL17, CCL22, CCL24, CXCR1 and Arg-1. In cancer, macrophage M2 polarization contributes to the growth of tumor cells and tumor metastasis. In our research, the mRNA expressions of macrophage M2 polarization cytokines were either not affected (Arg-1 and CCL17) or slightly decreased (CCL22) in rCMIP-treated macrophages (Figure 5C). All of these results combined to support the conclusion that CMIP exerts anti-metastasis activity through its immune activation activity on macrophages in our previous research. Additionally, these results also provide strong support for rCMIP as a potential macrophage activator in cases where the activation of macrophages should be induced.

## 4. Materials and Methods

### 4.1. Strains and Vectors

The fungus *C. militaris* was obtained from the Guangdong Institute of Microbiology (DIM, Guangzhou, China). The *Escherichia coli* strains, DH 5α and BL 21, were bought from Takara Biomedical Technology (Beijing, China). The *E. coli* vector (pET-30a (+)) was obtained from Invitrogen (Carlsbad, CA, USA). SA-FITC and the APC anti-mouse CD86 antibody were obtained from BioLegend (San Diego, CA, USA).

### 4.2. Materials and Chemicals

The TRIzol Reagent was purchased from Invitrogen (Carlsbad, CA, USA). The PolyATtract mRNA purification kit was bought from Promega (Madison, WI, USA). The Taq DNA polymerase and the restriction enzymes Nde I and Xho I were obtained from Takara Biomedical Technology (Beijing, China). The T4 DNA Ligase was bought from New England Biolabs (Beijing, China). The plasmid extraction and DNA gel extraction kits were purchased from BioTeke (Beijing, China). The tryptone, yeast extract, NaCl and other salts, agar and Tris base were purchased from Merck (Darmstadt, Germany). The IPTG and kanamycin were obtained from Yeasen Biotech (Hongkong, China). The EZ-Link NHS-Biotin reagents were from Thermo Scientific (Waltham, MA, USA).

### 4.3. Cloning of CMIP Gene

The fruiting body of *C. militaris* was washed thoroughly with distilled water and ground into powder in liquid nitrogen. Total mRNA was extracted using the PolyATtract mRNA purification kit according to the manufacturer’s instructions. The cDNA was synthesized using M-MLV Reverse Transcriptase (Promega, Madison, WI, USA). The immunoregulatory protein (CMIP) gene (Gene ID: CCM_01955) from *C. militaris* CM01 was amplified using specific primers which were designed according to the nucleotide sequence of CMIP. The sequences of specific primers were as follows: 5′-ATGGAGATCGCAGAACAGAACG-3′ (forward primer); 5′-CACGGTAATGAT CTTATAGTCGCC-3′ (reverse primer). The PCR conditions were listed as follows: pre-denaturation at 94 °C for 5 min, denaturation at 94 °C for 30 s, annealing at 60 °C for 30 s and extension at 72 °C for 1 min. The last three steps were repeated for 35 cycles and ultimately ended by one cycle of extension at 72 °C for 5 min. The PCR products were purified using E.Z.N.A. Cycle-Pure Kit (Omega Bio-tek, Norcross, GA, USA). The purified DNA fragment was directly inserted into a pET-30a vector through the restriction sites (Nde I and Xho I) and transformed into *E. coli* DH 5 cells. The grown colonies on an LB plate were sent to Sangon Biotech (Shanghai, China) for sequencing. The correct colonies were retained. The pET-30a_rCMIP plasmid was extracted from the DH 5 cells and transformed into *E. coli* BL 21 for further protein preparation.

### 4.4. Expression and Purification of Recombinant CMIP

The expression of the recombinant CMIP (rCMIP) was achieved using the pET-30a expression system (Invitrogen, Carlsbad, CA, USA). One clone with correct insertion grew in LB liquid medium containing 50 μg/mL of kanamycin at 37 °C overnight. Then, the culture was inoculated into LB liquid medium (1:100) containing ampicillin (50 μg/mL) and was cultured under the conditions given above to reach 0.6–0.8 optical density (OD) at 600 nm. Afterward, IPTG was added to the culture at a final concentration of 0.5 mM. Then, the mixture was incubated at 37 °C for 5 h. Finally, the cultured *E. coli* cells were harvested by centrifugation at 8000× *g* at 4 °C for 15 min. Pellet cells were collected, washed three times with PBS buffer and then sonicated. The lysate was centrifuged at 12,000× *g* at 4 °C for 20 min. The supernatant was collected for further purification.

Recombinant CMIP (rCMIP) was purified using the Ni^2+^-NTA column (Sangon Biotech, Shanghai, China). Briefly, after the column was equilibrated with PBS buffer, the supernatant prepared above was applied to the column. Subsequently, the column was washed with washing buffer. Finally, the target protein was eluted using an elution buffer. The flow rate was controlled at 1 mL/min for each step. The protein sample was thoroughly dialyzed against distilled water, then lyophilized and stored at −80 °C. The purified rCMIP was confirmed by 12% SDS–PAGE gel.

### 4.5. Biotinylation of rCMIP

rCMIP (3 mg/mL, 2 mL) was mixed with 10 mM of Sulfo-NHS-SS-Biotin (ThermoFisher Scientific, Waltham, MA, USA) solution and incubated on ice for 2 h. The non-reacted Sulfo-NHS-SS-Biotin was removed using a desalting column (Thermo Scientific, Waltham, MA, USA). The biotin-labelled rCMIP was collected and stored at −80 °C until use.

### 4.6. Cell Culture and Treatment

RAW 264.7 macrophages were purchased from the China Center for Type Culture Collection (CCTCC, Shanghai, China), and maintained at 5% CO_2_ atmosphere in DMEM medium supplemented with 10% (*v/v*) FBS, 100 U/mL of penicillin and 100 μg/mL of streptomycin. The medium was renewed every two days. The cells were inoculated into 6-well cultivation plates at an initial concentration of 3 × 10^5^ cells/well. After adhesion, the cells were treated with rCMIP at different concentrations according to test requirements. In all of the treatments, the endotoxin of recombinant CMIP (rCMIP) was removed with Pierce High Capacity Endotoxin Removal Spin Columns (ThermoFisher, Waltham, MA, USA) and the LPS contamination was <1 EU/mg.

### 4.7. Cell-Adhesion Assay, Annexin V/PI Staining and FACS Analysis

In the cell-adhesion assay, biotin-labeled rCMIP (0 μM and 2.5 μM) was incubated with RAW 264.7 macrophages at 4 °C for 2 h. After incubation, the cells were washed with PBS (pH 7.4) and exposed to SA-FITC for 30 min in the dark. For Annexin V/PI staining, RAW 264.7 cells were treated with rCMIP (0 μM, 1.6 μM and 3.2 μM) for 24 h. Then, the cells were collected, washed twice with PBS (pH 7.4), and stained using an Annexin V/PI kit (Multi Sciences Biotech, Hangzhou, China). All samples were analyzed using flow cytometry (CyAn ADP, Beckman, Fullerton, CA, USA) and data analysis was performed using CytExpert US (2.0 software).

### 4.8. Cell Proliferation Assay

RAW 264.7 cells were seeded at a concentration of 1 × 10^4^ cells/well. After adhesion, the cells were treated with CMIP (0 μM, 1.6 μM and 3.2 μM) for 24 h. The percentages of viable cells were determined using the CCK-8 kit (Dojindo, Kumamoto, Japan).

### 4.9. Quantitative Real-Time PCR for Cytokine mRNAs

The RAW 264.7 cells treated with rCMIP (0 μM, 0.32 μM and 1.6 μM) for 3 h were collected by centrifugation. Total RAN was extracted from the rCMIP-treated RAW 264.7 cells using TRIzol Reagent (Invitrogen, Carlsbad, CA, USA) according to the manufacturer’s instructions. The cDNA was synthesized from 1 μg of the total RNA preparation using M-MLV (Moloney Murine Leukemia Virus) Reverse Transcriptase (Promega, Madison, WI, USA). Quantitative real-time PCR was performed with SYBR Green Master on a Bio-Rad CFX96 system. The primers used were synthesized by Sangon Biotech (Shanghai, China) and the sequences are listed in Supplementary Table S1. The conditions for quantitative real-time PCR were listed as follows: initial denaturation at 95 °C for 10 min, followed by 40 cycles of denaturation at 95 °C for 10 s and annealing for 60 s. GAPDH was used as an endogenous control. Relative gene expression levels were analyzed by the 2^−ΔΔCT^ method.

### 4.10. Flow Cytometry Detection of CD86 Expression

The RAW 264.7 cells treated with rCMIP (0 μM, 0.64 μM and 1.6 μM) for 24 h were collected by gentle scraping using the PBS buffer and washed twice with the same buffer. Then, the cells were incubated with flow cytometry antibody APC CD86 (BioLegend, San Diego, CA, USA) on ice for 30 min. After staining, the cells were washed twice and then subjected to flow cytometry analysis.

### 4.11. iNOS Expression and NO Production Detection

To detect the iNOS protein expression, the RAW 264.7 cells treated with rCMIP (0 μM, 0.32 μM and 1.6 μM) for 24 h were collected. Total protein was extracted, quantified, separated by 12% SDS–PAGE and transferred to polyvinylidene fluoride (PVDF) membrane. The blotted membranes were incubated with 5% skim milk dissolved in TBST buffer at room temperature for 1 h. Subsequently, the membranes were incubated with anti-iNOS (catalog: A0312) and anti-GAPDH (catalog: A19056) antibody from ABclonal (Wuhan, China). After being washed, the membranes were incubated with HRP-conjugated goat anti-rabbit IgG (Proteintech, Wuhan, China) for 1 h. The membranes were washed, and the immunoblot was then developed with an ECL chemiluminescence detection kit (Thermo Scientific, Waltham, MA, USA) according to the manufacturer’s instructions. In the NO production assay, the culture supernatants of RAW 264.7 cells, which were treated with rCMIP (0 μM, 0.5 μM and 1.2 μM) for 24 h, were collected and the nitrite contents were determined by the Nitric Oxide Assay Kit (Beyotime, Shanghai, China). Briefly, 100 μL of supernatant was distributed in a 96-well plate and then equal volumes of the Griess reaction solutions were added. The mixture was incubated at room temperature for 10 min. The absorbance was read at 540 nm using a microplate reader. The nitrite concentration was determined from a sodium nitrite standard curve.

### 4.12. Statistical Analysis

All of the assays were performed in triplicate and the results were represented as the mean ± standard deviation (SD). Data were analyzed using GraphPad Prism software (version 5.04, GraphPad Software, USA). *p*-values less than 0.05 and less than 0.01 were considered to be statistically significant and highly significant, respectively.

## Supplementary Materials

The following are available online, Table S1: Primers for quantitative real-time PCR. Figure S1: Nucleotide sequences of CCM_01955 and its encoded amino acid sequence. Figure S2: Cell surface binding selectivity of natural purified CMIP.

## Sample Availability

Samples of the compounds are not available from the authors.
