# Supplementary material for: Molecular Cloning, Expression and Macrophage Activation of an Immunoregulatory Protein from Cordyceps militaris
Source: Molecules. 2021 Nov 24;26(23):7107. doi: 10.3390/molecules26237107 (PMC8658978; doi:10.3390/molecules26237107)
Supplement: Supplementary file 1 [file molecules-26-07107-s001.zip › molecules-1439965-supplementary.pdf]

Table S1. Primers for quantitative real-time PCR.

Gene	Forward primer	Reverse primer	Product Size (bp)
GAPDH	AGGTCGGTGTGAACGGATTTG	TGTAGACCATGTAGTTGAGGTCA	96
CCL2	TTAAAACCTGGATCGGAACCAA	GCATTAGCTTCAGATTTACGGGT	123
CCL4	TGCTCGTGGCTGCCTTCT	CTGCCGGGAGGTGAAGAGA	92
CCL5	ACTCCCTACTGCTTTGCCTAC	GCGGTTCCTTCGAGTGACA	121
CXCL10	GGAGTGAAGCCACGCACAC	ATGGAGAGGCTCTCTGCTGT	90
CXCL11	GGCTTCCTTATGTTCAAACAGGG	GCCGTTACTCGGGTAAATTACA	108
IL-1	TGCCACCTTTTGACAGTGATGA	AAGGTCCACGGGAAAGACAC	209
TNF-	CCCTCACACTCAGATCATCTTCT	GCTACGACGTGGGCTACAG	136
iNOS	CAGATCCCGAAACGCTTCA	TGTTGAGGTCTAAAGGCTCCG	190
IL-6	TAGTCCTCCCTACCCCAATTTCC	TTGGTCCTTAGCCACTCCTTC	109
Arg-1	GCCTTTGTTGATGTCCCTAATGA	CCACACTGACTCTTCCATTCTTC	110
CCL22	AGGTCCCTATGGTGCCAATGT	CGGCAGGATTTTGAGGTCCA	111
CCL17	TACCATGAGGTCACTTCAGATGC	GCACTCTCGGCCTACATTGG	312

CCM_01955	ATGGAGATCGCTGAGCAGAACGTTGGCAAGTCTTGTGCCATTGTCAAC	48
Protein sequence	M E I A E Q N V G K S C A I V N	
CCM_01955	CTCATCCCCCCAAAGGAGGGTAGCAACATCTGGACGGCCAAGCAGGTC	96
Protein sequence	L I P P K E G S N I W T A K Q V	
CCM_01955	ACCATTGCTCGGAACCTTCCTCCCAATCGTGGAACTGGCCGCCACCAG	144
Protein sequence	T I A R N L P P N R G T G R H Q	
CCM_01955	ATTGACTGGTCCTTTGGGCCCGTCAAGGTTACCGGTTATGTTGATACC	192
Protein sequence	I D W S F G P V K V T G Y V D T	
CCM_01955	ACCAACTGGGAGATTGGTGTGACTGTTAGCATCATGGGAATCGACCTT	240
Protein sequence	T N W E I G V T V S I M G I D L	
CCM_01955	GGCACCGTCTATGGAAACCTCAGAGACGGAGTCGTCCTCAACGTTGAC	288
Protein sequence	G T V Y G N L R D G V V L N V D	
CCM_01955	CTTTTCCTAGCCAAGGGCCAGGTCCGGTTCTACCTGAAGAACGGCAAC	336
Protein sequence	L F L A K G Q V R F Y L K N G N	
CCM_01955	GAAGTTTGGGTTCACCTGAGTGTTGAGATCAGATTCGACGGAAAGTTC	384
Protein sequence	E V W V H L S V E I R F D G K F	
CCM_01955	GAGGGCGACTACAAGATTATCACAGTTTAG	414
Protein sequence	E G D Y K I I T V *	

Figure S1. Nucleotide sequences of CCM_01955 and its encoded amino acid sequence.

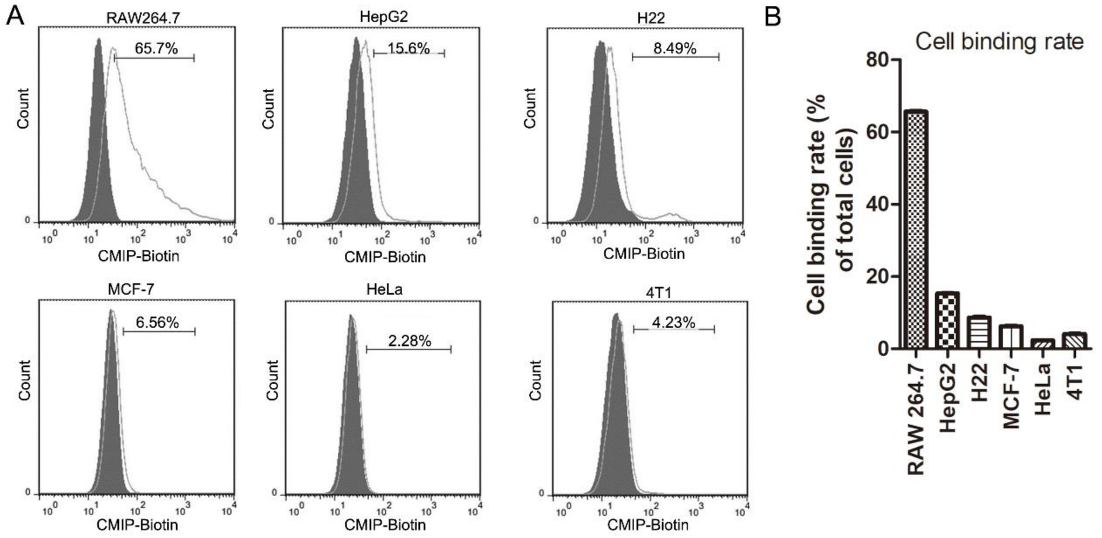

Figure S2. Cell surface binding selectivity of natural purified CMIP. (A) RAW264.7, HepG2, H22, MCF-7, HeLa and 4T1 cells (1 × 10^6) were incubated with biotinylated natural purified CMIP (2.5 M) at 4℃ for 2 h. After washing, cells were incubated with SA-streptavidin and analyzed by flow cytometry. Results are representative of three independent assays. (B) The cell labeling ratio quantitation of the tested cells from the flow cytometry results. All the data are presented with means±SD (n = 3) .
